# Analysis of the functional WT1-specific T-cell repertoire in healthy donors reveals a discrepancy between CD4^+^ and CD8^+^ memory formation

**DOI:** 10.1111/imm.12472

**Published:** 2015-06-19

**Authors:** Sabine Schmied, Emma Gostick, David A Price, Hinrich Abken, Mario Assenmacher, Anne Richter

**Affiliations:** 1 Miltenyi Biotec GmbHBergisch Gladbach, Germany; 2 Institute of Infection & Immunity, Cardiff University School of MedicineCardiff, UK; 3 Centre for Molecular Medicine Cologne, University of CologneCologne, Germany; 4 Department I Internal Medicine, University Hospital CologneCologne, Germany

**Keywords:** antigen-specific T cells, self-antigen, tumour-associated antigen, Wilms’ tumour-1 protein

## Abstract

The Wilms’ tumour-1 (WT1) protein is considered a prime target for cancer immunotherapy based on its presumptive immunogenicity and widespread expression across a variety of malignancies. However, little is known about the naturally occurring WT1-specific T-cell repertoire because self-derived antigens typically elicit low frequency responses that challenge the sensitivity limits of current detection techniques. In this study, we used highly efficient cell enrichment procedures based on CD137, CD154, and pHLA class I tetramer staining to conduct a detailed analysis of WT1-specific T cells from the peripheral blood. Remarkably, we detected WT1-specific CD4^+^ and CD8^+^ T-cell populations in the vast majority of healthy individuals. Memory responses specific for WT1 were commonly present in the CD4^+^ T-cell compartment, whereas WT1-specific CD8^+^ T cells almost universally displayed a naive phenotype. Moreover, memory CD4^+^ and naive CD8^+^ T cells with specificity for WT1 were found to coexist in some individuals. Collectively, these findings suggest a natural discrepancy between the CD4^+^ and CD8^+^ T-cell lineages with respect to memory formation in response to a self-derived antigen. Nonetheless, WT1-specific T cells from both lineages were readily activated *ex vivo* and expanded *in vitro*, supporting the use of strategies designed to exploit this expansive reservoir of self-reactive T cells for immunotherapeutic purposes.

## Introduction

The Wilms’ tumour-1 (WT1) protein is a transcription factor expressed at high levels in several haematological malignancies and some solid tumours. As a consequence of its oncogenic potential and presumptive immunogenicity, WT1 was ranked top in a list of tumour-associated antigens prioritized for cancer immunotherapy.^1^ Indeed, spontaneous T-cell responses against WT1 are induced in patients with leukaemia after allogeneic stem cell transplantation or donor lymphocyte infusions^2^–^4^ and are associated with disease regression.^4^ WT1-specific T cells may also be involved in cancer immune surveillance. However, little is known about the naturally occurring WT1-specific T-cell repertoire and its functional relevance *in vivo*, in part because of the exceedingly low frequencies of such cells in healthy donors. This knowledge gap potentially hinders the rational development of an effective WT1-directed T-cell therapy.

WT1-specific CD8^+^ T cells have been detected *ex vivo* in myeloid leukaemia patients by peptide-HLA class I (pHLAI) tetramer staining^5^ and by quantitative PCR for interferon-*γ* (IFN-*γ*) mRNA after antigenic stimulation, revealing elevated frequencies following stem cell transplantation.^6^,^7^ Highly sensitive IFN-*γ* mRNA analysis also provided the first hint that such cells exist in healthy individuals, although detailed characterization was precluded by technical constraints at the detection limit.^6^,^7^ Similar issues hamper the reliable detection of auto-reactive and tumour-associated antigen-specific T cells in healthy donors by other methods, including IFN-*γ* ELISpot analysis and pHLAI tetramer staining. As an exception, Melan-A/MART-1-specific CD8^+^ T cells can be detected at high frequencies in the naive repertoire of healthy individuals.^8^,^9^

To detect T cells specific for self-derived antigens other than Melan-A/MART-1 *ex vivo* in healthy donors, additional strategies must be employed to overcome the sensitivity limits of conventional methods. Magnetic enrichment of pHLAI tetramer^+^ cells has been implemented successfully in this regard to detect rare NY-ESO-1-specific CD8^+^ T cells^10^ and gp100-specific CD4^+^ T cells.^11^ In addition, surface molecules up-regulated after antigenic stimulation allow the visualization of activated antigen-specific T cells. For example, CD154 (CD40L) is expressed within a few hours after antigenic stimulation of CD4^+^ T cells. This approach has been used successfully in conjunction with antigen-specific enrichment to detect WT1-specific T cells *ex vivo* in healthy donors; the frequencies of these cells were calculated to range from 10^−6^ to 10^−5^ within the CD4^+^ T-cell compartment.^12^ The activation marker CD137 (4-1BB) further enables the detection of antigen-specific CD4^+^ and CD8^+^ T cells from the naive and memory pools.^13^ However, stimulation for more than 24 hr is required to induce CD137 on naive T cells, potentially distorting the phenotypic composition of activated cells acquired with this approach.

In this study, we used enrichment techniques based on pHLAI tetramer staining and the up-regulation of activation markers to characterize the entire WT1-specific T-cell repertoire functionally and phenotypically in a comprehensive and highly sensitive manner. Our approach incorporated multi-colour flow cytometric analysis directly *ex vivo* or after short-term *in vitro* expansion. Virtually all healthy donors harboured WT1-specific T cells in their peripheral blood. In the CD4^+^ cell compartment, memory T cells specific for WT1 were detected in 60% of cases. In contrast, WT1-specific CD8^+^ T cells retained a naive phenotype in the vast majority of donors. These findings highlight a natural discrepancy between the CD4^+^ and CD8^+^ T-cell lineages with respect to memory formation in response to a self-derived antigen.

## Materials and methods

### Isolation of peripheral blood mononuclear cells

Buffy coats or leukapheresis products were obtained from healthy donors at the University Hospital in Dortmund and Cologne. The study was performed according to established ethical guidelines and all blood donors gave informed consent. Peripheral blood mononuclear cells (PBMCs) were isolated using Ficoll–Hypaque (GE Healthcare, Chalfont St Giles, UK) density gradient centrifugation.

### Stimulation, isolation, and expansion of antigen-specific T cells

Freshly isolated PBMCs were resuspended in RPMI-1640 medium supplemented with 5% human AB serum (Lonza, Basel, Switzerland), 2 mm l-glutamine (GE Healthcare), and 1 µg/ml CD28 monoclonal antibody (mAb) at functional grade purity (Miltenyi Biotec, Bergisch Gladbach, Germany). Subsequently, 1 × 10^8^ PBMCs were stimulated at 1 × 10^7^ cells/ml with the optimal WT1_126_ peptide (RMFPNAPYL) and pooled WT1 15-mer peptides, overlapping by 11 amino acids, covering the whole WT1 isoform-1 protein sequence (Miltenyi Biotec); each peptide was present at a final concentration of 0·6 nmol/ml. Controls in the absence of exogenous peptide were included in all cases.

For *ex vivo* characterization of WT1-specific CD4^+^ T cells, PBMCs were stimulated for 7 hr in the presence of 1 µg/ml CD40 mAb at functional grade purity (Miltenyi Biotec). Brefeldin A (1 µg/ml; Sigma-Aldrich, St Louis, MO) was added 2 hr before harvest. CD154^+^ cells were isolated by indirect magnetic labelling using CD154-allophycocyanin and anti-allophycocyanin-MicroBeads (Miltenyi Biotec). Samples were loaded on MS Columns (Miltenyi Biotec) and intracellular staining was performed directly in the magnetic field using an Inside Stain Kit (Miltenyi Biotec). Frequencies of WT1-specific CD4^+^ T cells were calculated by subtracting the mean number of CD4^+^ CD154^+^ cells (triplicates) obtained after enrichment of non-stimulated PBMCs from the mean number of CD4^+^ CD154^+^ cells (triplicates) obtained after enrichment of WT1 peptide-stimulated PBMCs and dividing by the total number of CD4^+^ T cells applied to the column. A positive WT1-specific response was recorded if the mean CD4^+^ CD154^+^ count after WT1 peptide pool stimulation exceeded the 95% confidence interval for the CD4^+^ CD154^+^ count in the unstimulated sample (mean plus two times the standard error of the mean).

Antigen-reactive CD4^+^ and CD8^+^ T cells were isolated after 24–36 hr using a CD137 MicroBead Kit (Miltenyi Biotec) and expanded in X-Vivo15 (Lonza) supplemented with 5% human AB serum (Lonza), 5 ng/ml recombinant interleukin-7 (rIL-7), 5 ng/ml rIL-15, and 10 ng/ml rIL-21 (all Miltenyi Biotec). The corresponding CD137^−^ cell fractions were depleted of CD3^+^ cells using CD3-MicroBeads (Miltenyi Biotec), treated with mitomycin C (Sigma-Aldrich), and co-cultured as peptide-loaded autologous antigen-presenting cells (APCs) with activated T cells at a ratio of 100 : 1 with a final density of 5 × 10^6^ cells/ml in 24-well plates. Medium, including the recombinant cytokines, was replenished every second day. Cells were washed and counted on day 6 and then expanded for a further 3 days.

### Re-stimulation of expanded antigen-specific T cells

Two days before re-stimulation, cells were washed and plated at a concentration of 2·5 × 10^6^ cells/ml in cytokine-free medium. Thawed autologous CD3-depleted PBMCs were pulsed with pooled peptides (0·6 nmol/ml/peptide) overnight, washed once, and co-cultured as APCs at a ratio of 1 : 5 with expanded T cells in RPMI-1640 medium supplemented with 5% human AB serum and 1 µg/ml CD28 mAb at functional grade purity in 96-well plates for 6 hr. Brefeldin A (1 µg/ml) was added after 2 hr of co-culture. For assessment of degranulation, co-cultures were supplemented with CD107a-PE (phycoerythrin; Miltenyi Biotec) and 1 µg/ml monensin (eBioscience, San Diego, CA). Cells were then fixed, permeabilized, and stained intracellularly using a Rapid Cytokine Inspector (Miltenyi Biotec).

For determination of functional avidity, T2 cells were pulsed overnight with escalating doses of the optimal WT1_37_ peptide (VLDFAPPGA) and co-cultured with expanded T cells at a ratio of 1 : 25 before staining as described above. For measurement of direct cytotoxicity, T2 cells were either pulsed with peptide pools (specific: WT1; unrelated: HPV16 E7; Miltenyi Biotec) or left unpulsed overnight, labelled with different concentrations of VioDye (Invitrogen, Carlsbad, CA) for 5 min at 37°C, washed three times, and pooled. Labelled T2 cell pools were then added to expanded T cells at different effector to target (E : T) ratios for 4 hr. For blocking experiments, 1 µg/ml anti-MHCI mAb (W6/32) was added to the co-culture. Antigen-specific killing was determined by flow cytometric measurement of T2 cell viability. Percentage killing was calculated by subtracting the ratio of living antigen-loaded T2 cells after co-culture in the presence or absence of T cells from unity. Percentage antigen-specific killing was calculated by subtracting killing determined for non-loaded T2 cells from killing determined for antigen-loaded T2 cells.

### Flow cytometry

The following mAbs were used for staining and flow cytometric measurements: CD3-VioGreen (BW264/56), CD4-allophycocyanin-Vio770 (VIT4), CD8-PE-Vio770 (BW135/80), CD14-peridinin chlorophyll protein (PerCP) (TÜK4), CD20-PerCP (LT20), CD27-FITC (M-T271), CD28-FITC (15E8), CD45RA-VioBlue (T6D11), CD45RO-FITC (UCHL1), CD62L-FITC (145/15), CD107a-PE (1D4B), CD127-FITC (MB15-18C9), CD137-allophycocyanin, CD137-PE (4B4-1), CD154-VioBlue (5C8), CD178-PE (NOK-1), CD197 (CCR7)-allophycocyanin, CD197 (CCR7)-PE (150503), CD279-allophycocyanin (PD1.3.1.3), anti-IL-2-PE-Vio770 (N7.48A), anti-IL-4-PE (7A3-3), anti-IL-17-FITC (CZ8-23G1), anti-IFN-*γ*-FITC, anti-IFN-*γ*-PE-Vio770 (45-15), anti-TNF-*α*-PE, and anti-TNF-*α*-VioBlue (cA2) (all Miltenyi Biotec).

Soluble biotinylated pHLA-A*0201 molecules loaded with WT1_37_ (VLDFAPPGA), WT1_126_ (RMFPNAPYL), WT1_187_ (SLGEQQYSV), WT1_235_ (CMTWNQMNL), or cytomegalovirus (CMV) pp65_495_ (NLVPMVATV) were produced as described previously.^14^ Tetramerization was achieved by binding to streptavidin-PE or streptavidin-allophycocyanin (both BioLegend, San Diego, CA). In each case, 2·5 × 10^6^ cells were stained with 1 µg/ml tetramer for 45 min at 4°C.

Data were acquired using a MACSQuant flow cytometer and analysed with MACSQuantify software (both Miltenyi Biotec).

### Sorting of T-cell subsets

Freshly isolated PBMCs from leukapheresis products were used for all T-cell subset purifications. Naive T cells were sorted at > 99% purity using a human Naive Pan T Cell Isolation Kit (Miltenyi Biotec). Memory T cells were sorted using a human Pan T Cell Isolation Kit (Miltenyi Biotec) and additionally depleted of CD45RA^+^ cells using CD45RA-MicroBeads (Miltenyi Biotec). Sorted T cells (1 × 10^8^) were supplemented with 1 × 10^7^ APCs and pooled peptides for stimulation.

### Enrichment of tetramer^+^ cells

For antigen-specific T-cell enrichment, 1 × 10^8^ Pan T cells were labelled with PE-conjugated WT1_37_ tetramer (0·5 µg/ml) for 15 min at 37°C. Allophycocyanin-conjugated WT1_37_ tetramer (1 µg/ml) and mAbs were then added for 15 min at 4°C. After washing, cells were incubated with anti-PE-MicroBeads (Miltenyi Biotec) for 15 min at 4°C and washed again. Separation was performed using two subsequent MS columns according to the manufacturer's protocol (Miltenyi Biotec). The positive fraction was analysed directly using a MACSQuant flow cytometer (Miltenyi Biotec). Cells were gated hierarchically as follows: lymphocytes, singlets, viable cells, CD14^−^ and CD20^−^, CD3^+^, and CD8^+^. Frequencies of WT1_37_-specific CD8^+^ T cells were determined by dividing the number of WT1_37_ tetramer-PE/allophycocyanin double-positive CD8^+^ T cells by the number of total CD8^+^ cells applied to the column.

### Statistics

The paired Student's *t*-test was used to assess significance. Analyses were conducted using Prism software version 6.0 (GraphPad, San Diego, CA).

## Results

### Functionally competent WT1-specific T cells can be efficiently propagated *in vitro* from healthy donors

To assess WT1-specific T cells in the peripheral blood of healthy donors, we stimulated PBMCs with a WT1 peptide mix for 24–36 hr and subsequently enriched CD137^+^ cells. CD137 (4-1BB) is up-regulated upon T-cell receptor (TCR) engagement and was previously used to detect antigen-specific memory and naive CD4^+^ and CD8^+^ T cells.^13^,^15^

Flow cytometric analysis of the enriched cell fractions revealed significantly elevated numbers of CD4^+^ CD137^+^ and CD8^+^ CD137^+^ T cells in WT1-stimulated PBMC samples compared with unstimulated controls (see Supplementary material, Fig. S1). Absolute cell numbers after enrichment ranged from 1·2 × 10^4^ to 4·3 × 10^4^ (mean 3·3 × 10^4^) viable CD137^+^ leucocytes in the stimulated samples compared with 0·9 × 10^4^ to 3·2 × 10^4^ (mean 2·5 × 10^4^) cells in the control samples (see Supplementary material, Fig. S1b). This direct numeric comparison suggests that the majority of enriched T cells in the WT1-stimulated samples were pre-activated *in vivo* by antigens other than WT1. To demonstrate the presence of WT1-reactive T cells in the CD137^+^ fraction and obtain sufficient cell numbers for further characterization, we expanded the enriched cell populations for 9 days *in vitro* (see Supplementary material, Fig. S1c).

**Figure 1:**
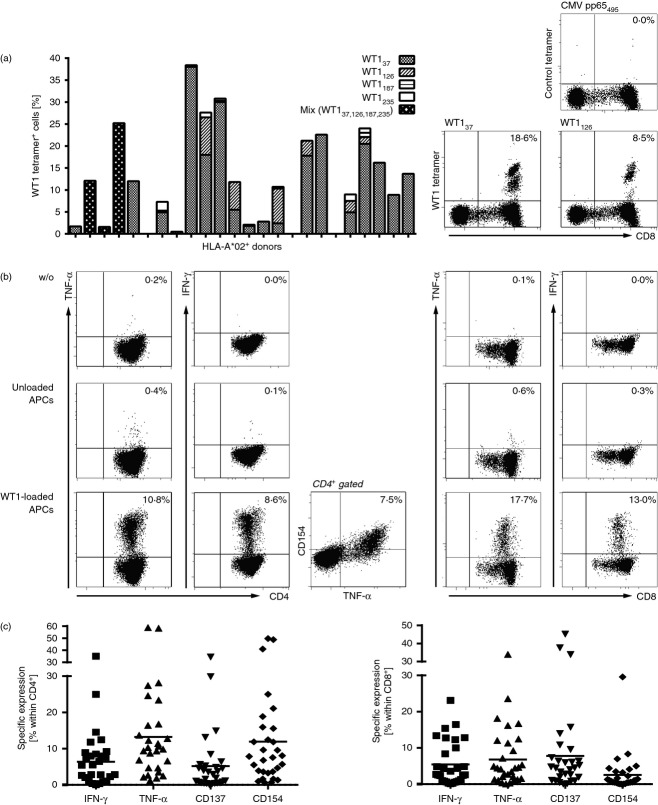
Functionally competent, oligoclonal Wilms’ tumour-1 (WT1)-specific CD4^+^ and CD8^+^ effector T cells can be generated from healthy donors. Peripheral blood mononuclear cells (1 × 10^8^) were stimulated with pooled, overlapping WT1 15-mer peptides for 24–36 hr, enriched for CD137^+^ cells, and expanded for 9 days. (a) Frequencies of WT1 tetramer^+^ cells specific for four different HLA-A*0201-restricted epitopes within the CD8^+^ T-cell compartment (*n* = 24; 10 independent experiments were performed; n.d., not detected). Dot plots show representative stainings of expanded T cells from one donor with the indicated tetramers (CMV pp65_495_ was used as a negative control). (b, c) Intracellular staining for interferon-*γ* (IFN-*γ*), tumour necrosis factor-*α* (TNF-*α*), CD137, and CD154 expression after re-stimulation of expanded T-cell cultures for 6 hr with autologous unloaded antigen-presenting cells (APCs) or APCs loaded with WT1 peptides. (b) Dot plots show representative stainings of one T-cell culture. Frequencies within the CD4^+^ or CD8^+^ T-cell compartment are depicted. (c) Frequencies of CD4^+^ and CD8^+^ T cells that produce cytokines or express activation markers after re-stimulation (*n* = 30; 12 independent experiments were performed). T-cell frequencies after co-incubation with unloaded APCs were subtracted. Bar represents mean.

Tetramer staining confirmed the antigen specificity of expanded T cells in the CD8^+^ compartment (Fig.1a). In 21/24 HLA-A2^+^ samples, WT1-specific cells were detected by at least one of four pHLA-A*0201 tetramers based on the previously described epitopes WT1_37_, WT1_126_, WT1_187,_^16^ and WT1_235._^17^ The WT1_37_ epitope was both immunodominant (0·5–30% within CD8^+^) and immunoprevalent (18/24 samples). Moreover, in 10/18 samples with WT1_37_ reactivity, at least one additional tetramer^+^ population was recorded. Further WT1 specificities may exist within the expanded repertoire, including reactivities beyond the context of HLA-A*0201. Notably, two specific populations with heterogeneous staining intensities were observed with one tetramer in some cases. These data imply differential avidities for cognate antigen, potentially reflecting either distinct clonotype usage or alternative activation states.^18^,^19^

Functional characterization of the expanded T-cell fractions further confirmed their specificity for WT1. After short-term re-stimulation with WT1 peptide pool-loaded autologous APCs, substantial proportions of WT1-reactive T cells produced IFN-*γ* and tumour necrosis factor-*α* (TNF-*α*) and expressed the activation markers CD137 and CD154 (Fig.1b,c). Minimal responses were observed with control APCs, although IFN-*γ* production by CD4^+^ T cells occurred at frequencies up to 4·8% in 12/30 donors. Antigen-specific cytokine production was detected in all cultures obtained from healthy donors after stimulation. Mean frequencies for TNF-*α* production were 12·4% for CD4^+^ T cells (range 2–59%) and 6·4% for CD8^+^ T cells (range 0·5–34%). The corresponding frequencies for IFN-*γ* production were 6·0% (range 0·5–36%) and 5·1% (range 0·5–23%) of T cells, respectively. Each donor harboured lymphocytes with a WT1-induced cytokine response in the CD4^+^ and/or CD8^+^ T-cell compartment.

Cytokine production correlated with activation marker expression. Mean frequencies for WT1-induced CD137 up-regulation were 4.9% for CD4^+^ T cells (range 0.5–35%) and 7.3% for CD8^+^ T cells (range 0.5–45%). The corresponding frequencies for CD154 expression were 11·2% (range 1·5–50%) and 2·4% (range 0·2–30%), respectively. CD154 expression levels on CD8^+^ T cells were low compared with cytokine production. This observation is consistent with previous reports in the context of CD4^+^ T cells.^20^ Notably, WT1-specific cytokine production was also detected in all three donors lacking tetramer^+^ populations.

Expanded WT1-specific T cells mobilized CD107a after antigen encounter, indicating antigen-induced degranulation and lytic potential (see Supplementary material, Fig. S2a). This was also marked for CD4^+^ T cells, consistent with previous reports.^21^ Direct cytotoxicity against WT1 peptide-loaded T2 cells was also recorded for CD8^+^ T cells (see Supplementary material, Fig. S2b), indicating the potential for a WT1-driven cytolytic attack.

Phenotypic analyses after short-term expansion showed that the majority of tetramer^+^ WT1-specific T cells expressed CD27, CD28, and CD127 within a predominant CD45RO^+^ CCR7^−^ effector memory phenotype. These cells did not express CD57, CD178, or CD279, implying a lack of senescence and exhaustion.

Collectively, these data show that WT1-reactive CD4^+^ and CD8^+^ T cells are present in the peripheral blood of virtually all healthy donors. Furthermore, these cells are amenable to expansion while retaining their functional and phenotypic assets *in vitro*.

### WT1-reactive memory CD4^+^ T cells are present in the majority of healthy donors

To enumerate and characterize rare, pre-existing WT1-specific CD4^+^ T cells in healthy donors directly *ex vivo*, we used a previously reported antigen-reactive T-cell enrichment (ARTE) protocol.^12^ In brief, the approach enables the analysis of antigen-specific CD4^+^ T cells at frequencies as low as 10^−6^ by magnetic enrichment of CD154^+^ cells after stimulation of 10^7^–10^8^ PBMCs. As CD154 is up-regulated within a few hours after TCR ligation, it allows simultaneous quantification and functional characterization of the antigen-reactive CD4^+^ T-cell pool.

**Figure 2:**
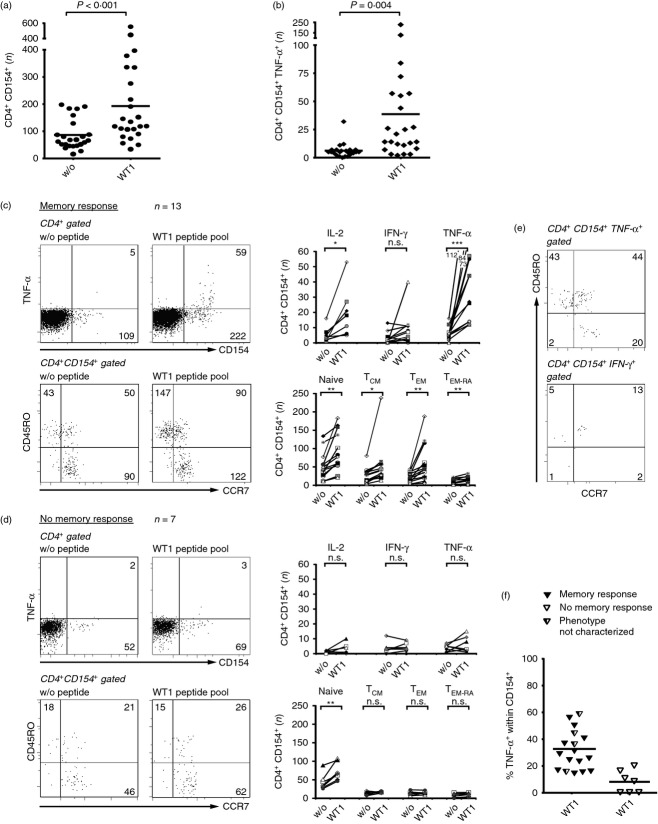
Wilms’ tumour-1 (WT1)-specific memory CD4^+^ T cells are detected in the majority of healthy donors. Peripheral blood mononuclear cells were incubated with or without pooled WT1 peptides for 7 hr and then enriched for CD154^+^ cells. (a) Cell count (*n*) of CD4^+^ CD154^+^ cells after enrichment of CD154^+^ cells in unstimulated (w/o) and WT1-stimulated (WT1) samples (*n* = 24; means of at least two technical replicates are depicted; 12 independent experiments were performed). Bar represents mean; significance was determined using a paired Student's *t*-test. (b) Cell count (*n*) of CD4^+^ CD154^+^ TNF-*α*^+^ cells after enrichment of CD154^+^ cells in unstimulated (w/o) and WT1-stimulated (WT1) samples. Bar represents mean; significance was determined using a paired Student's *t*-test. (c, d) Dot plots (left) and enumeration (right) of flow cytometric stainings for tumour necrosis factor-*α* (TNF-*α*) and CD4^+^ CD154^+^ cell phenotype. Enriched CD4^+^ CD154^+^ cells, subdivided phenotypically into CD45RO^−^ CCR7^+^ naive, CD45RO^+^ CCR7^+^ central memory (T_CM_), CD45RO^+^ CCR7^−^ effector memory (T_EM_), and CD45RO^−^ CCR7^−^ terminally differentiated effector (T_EM__-__RA_) T cells or functionally according to cytokine expression were enumerated in unstimulated (w/o) and WT1-stimulated (WT1) samples. Total cell counts obtained from 5 × 10^7^ peripheral blood mononuclear cells for donors with (c) or without (d) a memory CD4^+^ T-cell response are shown. Significance was determined using a paired Student's *t*-test and defined as: *P < 0·05, **P < 0·01, ***P ≤ 0·001, n.s. = not significant. (e) Dot plots show a representative staining of WT1-stimulated cells producing TNF-*α* and interferon-*γ* (IFN-*γ*) from the blood of a single donor with a memory response against WT1 (*n* = 3 donors). (f) Frequencies of TNF-*α*-producing cells within WT1-reactive CD4^+^ CD154^+^ cells (*n* = 24; 12 independent experiments were performed). Background cell counts detected in the corresponding unstimulated samples were subtracted.

Numbers of CD4^+^ CD154^+^ T cells increased substantially after stimulation of PBMCs with WT1 peptides (mean 190 cells, range 50–555 cells) compared with unstimulated control samples (mean 87 cells, range 27–198 cells) in 22/24 donors (Fig.2a). On the basis of the WT1-specific CD154^+^ cell count after stimulation and the overall number of CD4^+^ T cells in each sample, WT1-reactive T-cell frequencies were calculated to range between 2 × 10^−6^ and 2 × 10^−5^ within the CD4^+^ T-cell compartment.

Intracellular TNF-*α* staining of enriched CD154^+^ cells was performed directly in a magnetic field to minimize cell loss^12^ (Fig.2b). WT1-induced TNF-*α*-producing CD4^+^ T cells were detected in 21/24 samples (3–228 CD154^+^ TNF-*α*^+^ cells in WT1-stimulated samples versus 0–32 CD154^+^ TNF-*α*^+^ cells in unstimulated controls). Frequencies ranged between 2 × 10^−7^ and 1 × 10^−5^ within the CD4^+^ T-cell compartment.

As these results imply considerable individual variation in the frequencies of detectable WT1-reactive CD154^+^ and CD154^+^ TNF-*α*^+^ cells, we conducted a more detailed phenotypic and functional analysis. Specifically, we evaluated CD45RO, CD45RA, and CCR7 expression in one sample along with TNF-*α* and IFN-*γ* production in a parallel sample from the same donor. In some individuals, we also evaluated the production of IL-2, IL-4, IL-10, and IL-17. Based on the phenotypic data, we divided the 20 analysed donors into two groups: (i) those with WT1-reactive naive and memory cells (*n* = 13); and (ii) those with WT1-reactive naive cells only (*n* = 7).

In the ‘memory response’ group, higher numbers of naive (CD45RO^−^ CCR7^+^) and memory (CD45RO^+^CCR7^+/−^) CD154^+^ T cells were present after WT1 stimulation compared with the corresponding unstimulated samples (Fig.2c). Moreover, at least 1·5-fold higher numbers of CD154^+^ cells were detected after enrichment in the WT1-stimulated samples compared with unstimulated controls. Ten or more CD154^+^ TNF-*α*^+^ events were detected in these samples. The frequency of TNF-*α*^+^ cells within the CD154^+^ compartment was generally higher compared with the ‘no memory response’ group (mean 29·6%, range 14·1–53·6%) (Fig.2f). The memory phenotype of cytokine-producing WT1-specific T cells was confirmed by co-staining for CD45RO, CCR7, TNF-*α*, and IFN-*γ* in three additional healthy donors (Fig.2e). Although WT1-specific TNF-*α*-producing cells predominantly expressed CD45RO, functional cells with a naive phenotype were also detected. In contrast, IFN-*γ* producers resided exclusively in the memory compartment. Notably, IL-2-producing cells were detected in 5/7 donors, IFN-*γ* production was detected in 5/14 donors and IL-17 production was detected in 1/7 donors.

In the ‘no memory response’ group, small numbers of WT1-specific CD154^+^ TNF-*α*^+^ cells were present (2 to 8 CD154^+^ TNF-*α*^+^ events above background) in 4/7 donors. All CD154^+^ events recognized above background displayed a naive CD45RO^−^ CCR7^+^ phenotype (Fig.2d). No WT1-induced production of IL-4 or IL-10 was observed. WT1-specific CD8^+^ CD154^+^ T cells were not detected (data not shown).

These data confirm that functional WT1-specific CD4^+^ T cells are present in all healthy donors and further demonstrate that the majority (> 60%) of healthy donors harbour a memory CD4^+^ T-cell pool specific for WT1.

### WT1-specific CD8^+^ T cells derive predominantly from the naive pool in healthy donors

**Figure 3:**
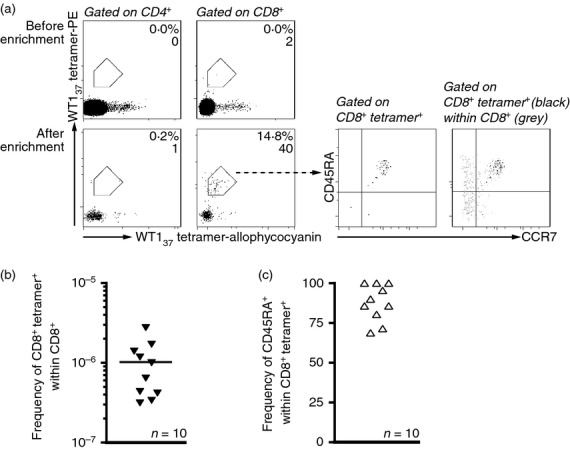
The majority of WT1_37_-specific CD8^+^ T cells exhibit a naive phenotype. WT1_37_ tetramer^+^ cells were enriched from 0·8 × 10^8^ to 1·2 × 10^8^ T cells using anti-phycoerythrin-MicroBeads. (a) Dot plots show representative enrichment of WT1_37_ tetramer^+^ cells. Dual staining with WT1_37_ tetramers is shown for CD8^+^ and CD4^+^ cells. Percentages within the CD8^+^ T-cell compartment and total counts of double-positive cells are shown. Stainings for CD45RA and CCR7 are shown for CD8^+^ tetramer^+^ cells and all CD8^+^ cells. (b) Frequencies of CD8^+^ tetramer^+^ cells within the CD8^+^ compartment for 10 HLA-A2^+^ donors (6 independent experiments were performed). (c) Frequencies of CD45RA^+^ cells within WT1_37_-specific CD8^+^ tetramer^+^ cells (*n* = 10; 6 independent experiments were performed).

To investigate the *ex vivo* phenotype of WT1-specific CD8^+^ T cells, we performed magnetic enrichment of WT1_37_ tetramer^+^ cells directly from HLA-A2^+^ healthy donor-derived PBMCs. The WT1_37_ epitope was chosen for these experiments as this epitope is the most abundant specificity after CD137 enrichment (Fig.1a). Across 16 samples, CD8^+^ tetramer^+^ events ranged from 1 to 55 cells (mean 14 cells). Two internal control mechanisms were employed to eliminate non-specific background staining.^22^ First, dual labelling was performed with the WT1_37_ tetramer conjugated to allophycocyanin or PE. Enrichment was then performed using anti-PE-MicroBeads and only double-positive events were recorded as antigen-specific. Second, concomitant staining of CD4^+^ T cells was used as a reference standard (Fig.3a). For enumeration and phenotypic analysis, we included samples with at least five CD8^+^ tetramer^+^ events (*n* = 10). Using this approach, frequencies of WT1_37_-specific T cells ranged from 3 × 10^−7^ to 3 × 10^−6^ (mean 1 × 10^−6^) within the CD8^+^ T-cell compartment (Fig.3b). Moreover, the vast majority of these WT1_37_-specific CD8^+^ T cells (70–100%) displayed a naive CD45RA^+^ CCR7^+^ phenotype (Fig.3a,c). We conclude that the majority of WT1_37_-specific CD8^+^ T cells detected in healthy donors emanate from the naive pool.

### Phenotypic disparity between CD4^+^ and CD8^+^ T cells specific for WT1

*Ex vivo* characterization of WT1-reactive CD4^+^ T cells using the ARTE protocol revealed a high proportion of donors with antigen-specific memory responses in the periphery. In contrast, the majority of WT1-specific CD8^+^ T cells detected *ex vivo* by WT1_37_ tetramer enrichment exhibited a naive phenotype.

**Figure 4:**
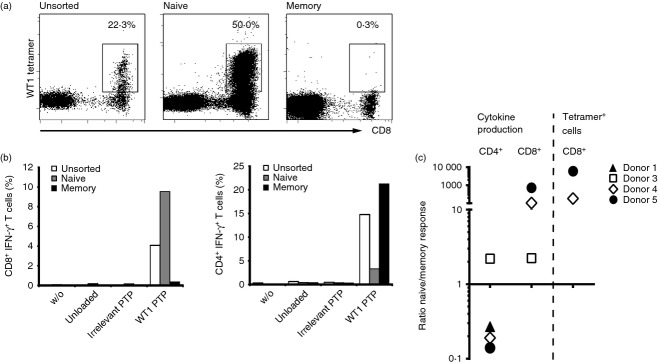
Phenotypic disparity between CD4^+^ and CD8^+^ T cells specific for Wilms’ tumour-1 (WT1). Presorted naive (CD45RA^+^ CCR7^+^) or memory (CD45RO^+^) T cells or unsorted peripheral blood mononuclear cells (1 × 10^8^ cells each) were stimulated for 36 hr, enriched for CD137^+^ cells, and expanded. (a) Representative analysis (donor 4) of WT1 tetramer^+^ cells after expansion of T cells obtained from presorted (naive/memory) or unsorted peripheral blood mononuclear cells. Cells were stained with a mixture of HLA-A*0201 tetramers loaded with four different WT1 epitopes. Plots are gated on CD3^+^ events. Percentages of tetramer^+^ cells within the CD8^+^ T-cell compartment are shown. (b) Intracellular cytokine staining after re-stimulation of expanded cells from a representative individual (donor 4) using either unloaded or differentially loaded autologous antigen-presenting cells (PTP, peptide pool). (c) Summary of WT1-specific responses for four different donors. Ratios of naive/memory-derived responses were calculated for WT1-induced tumour necrosis factor-*α* (TNF-*α*)-producing CD4^+^ and CD8^+^ T cells after re-stimulation as well as CD8^+^ tetramer^+^ T cells.

To evaluate the *in vivo* phenotypic origin of WT1-specific CD8^+^ T cells across further specificities, we pre-sorted T-cell subsets before stimulation. This approach allows the simultaneous detection of WT1-reactive CD4^+^ and CD8^+^ T cells and determination of their phenotype in one sample. Naive CD45RA^+^ CCR7^+^ and memory CD45RO^+^ T cells were sorted by magnetic cell separation in an untouched manner from five sets of donor PBMCs. In all cases, subset purity was > 99% within the CD3^+^ compartment (data not shown). Subset-defined T cells (0·5 × 10^8^ to 1 × 10^8^) were then stimulated with pooled WT1 peptides and autologous CD3-depleted APCs for 36 hr, sorted on the basis of CD137 expression and expanded. Tetramer analysis was used to evaluate the HLA-A2^+^ samples (donors 2, 4, and 5). In all three cases, tetramer^+^ cells were detected in T-cell cultures derived from the naive pool but not from the memory pool, suggesting that WT1_37_-specific CD8^+^ T cells emanate from the naive repertoire in healthy donors (Fig.4a). WT1-specific CD8^+^ T cells were also recorded by staining with mixed tetramers in the unsorted fractions, which were stimulated and enriched for CD137^+^ cells. No tetramer^+^ cells were detected in either T-cell fraction without previous enrichment due to methodological sensitivity limitations. Collectively, these data support the conclusion that WT1-reactive CD8^+^ T cells exhibit a naive phenotype in healthy donors.

Pre-sorting T-cell subsets further allowed side-by-side phenotypic comparisons of WT1-specific CD4^+^ and CD8^+^ T cells in individual donors. Expanded T cells were antigen-specific in four cases (donors 1, 3, 4, and 5) (Fig.4b). Again, WT1-reactive CD8^+^ T cells were detected in samples derived from the naive pool but not from the memory pool (Fig.4b), confirming the results obtained by tetramer analysis (Fig.4a). However, WT1-reactive CD8^+^ T cells in donor 3 emerged from both the naive and memory pools, representing an exception to the general pattern (Fig.4c). In contrast to CD8^+^ T cells, the highest frequencies of WT1-reactive CD4^+^ T cells derived from the memory pool (Fig.4b,c), confirming the *ex vivo* phenotypic analyses (Fig.2). WT1-specific CD4^+^ and CD8^+^ T cells were also detected in the unsorted fractions (Fig.4b).

Taken together, these results confirm the presence of WT1-reactive CD4^+^ and CD8^+^ T cells in the memory and naive pools, respectively. It is notable that these distinct phenotypically defined WT1-specific T-cell populations can coexist in the same individual.

## Discussion

In this study, we used cell enrichment strategies to conduct an in-depth analysis of the physiological WT1-specific T-cell repertoire in healthy individuals. The key findings were: (i) WT1-specific T cells are present in the vast majority of healthy donors; (ii) WT1-specific CD4^+^ T cells are commonly present in the memory pool; (iii) WT1-specific CD8^+^ T cells reside primarily in the naive pool; and (iv) WT1-specific memory CD4^+^ and naive CD8^+^ T cells can coexist in the same individual. Collectively, these data imply that distinct mechanisms control the differentiation of auto-reactive WT1-specific CD4^+^ and CD8^+^ T lymphocytes.

The enrichment of WT1-reactive T cells in this study was based on CD137 up-regulation after *in vitro* stimulation with pooled WT1 peptides, which results in an oligoclonal amplification of WT1-reactive T cells. In these short-term cultures, both CD4^+^ and CD8^+^ T cells produced pro-inflammatory cytokines in response to WT1 peptides, providing evidence for functional WT1-specific immunity in healthy donors.

The abundance of functional WT1-reactive CD4^+^ T cells was confirmed by enrichment of CD154^+^ T cells after short-term antigen-specific stimulation. Based on this analysis, the calculated prevalence of WT1-specific T cells ranged from 10^−6^ to 10^−5^ in the CD4^+^ cell compartment. These numbers are consistent with published data for WT1-specific CD4^+^ T cells quantified using the same procedure^12^ and for CD4^+^ T cells that recognize other self-derived or neo-antigens, including gp100, fibrinogen, preproinsulin^11^ and *Bacillus anthracis*^23^, detected by pHLAII tetramer enrichment. In addition, the ARTE assay enabled direct *ex vivo* characterization of WT1-specific CD4^+^ T cells in terms of function and phenotype. Based on this approach, we estimated the presence of WT1-reactive memory CD4^+^ T cells in > 60% of healthy donors. These WT1-specific memory CD4^+^ T cells readily produced TNF-*α* and IFN-*γ* in response to WT1 peptide-loaded APCs.

In the CD8^+^ T-cell compartment, calculated frequencies of WT1-specific T cells ranged from 10^−7^ to 10^−5^ based on pHLAI tetramer enrichment studies. Although these numbers are nearly one order of magnitude below those estimated for WT1-specific CD4^+^ T cells, it is noteworthy that our approach covered only the WT1_37_ epitope rather than all potentially antigenic regions of the WT1 protein. Moreover, the frequencies of WT1_37_-specific CD8^+^ T cells reported here are similar to those detected previously for other self-derived or non-encountered viral antigens, including NY-ESO-1 (10^−6^ to 10^−5^) and HIV-1 (10^−7^ to 10^−5^).^10^

Our work provides the first direct comparison of CD4^+^ and CD8^+^ T cells with the same self-derived antigen specificity. In this regard, we detected a profound phenotypic discrepancy between WT1-specific CD4^+^ and CD8^+^ T cells after sorting naive and memory T-cell subsets. WT1-specific CD4^+^ T cells were identified in the memory subset, whereas WT1-specific CD8^+^ T cells arose solely from the naive pool. This dichotomy contrasts with the classical model of antigen-specific T-cell memory formation based on cognate antigen encounter. Alternative mechanisms invoking T-cell homeostasis or cross-reactivity with other antigens may therefore come into play. On one side, antigen-specific CD8^+^ T-cell populations with memory-like properties can be generated in unexposed mice by homeostatic expansion.^24^,^25^ On the other side, virus-specific CD4^+^ T cells in unexposed humans can be driven into the memory pool as a consequence of cross-reactivity with common environmental antigens.^11^ It is also pertinent to note that low-avidity naive cells might not be driven into memory and that self-derived antigen-specific T-cell priming can be inhibited by regulatory T cells.^26^,^27^

Our data imply that memory formation is more common for WT1-reactive CD4^+^ T cells than for CD8^+^ T cells with the same antigen specificity. Although cross-reactivity clearly exists within the CD8^+^ T-cell compartment^28^–^30^, we speculate that this phenomenon is the driving mechanism that generates WT1-specific memory CD4^+^ T cells. Cross-reactivity may accordingly be more common in the CD4^+^ T-cell compartment and/or the relevant antigens that trigger this process may be more prevalent for CD4^+^ T cells.^31^,^32^ Indeed, an *in silico* database search identified presumed CD4^+^ T-cell epitopes derived from common pathogens that might induce such cross-reactivity (see Supplementary material, Fig. S3).

In support of our hypothesis, two observations challenge the concept of ‘virtual memory’ in this instance. First, WT1-specific CD4^+^ T cells readily produced effector cytokines in response to cognate antigen encounter. Second, memory cells represented a major subset of the total WT1-specific CD4^+^ T-cell population in all healthy donors. In contrast, the virtual memory T-cell repertoire typically comprises < 20% of the corresponding antigen-specific population.^24^,^25^

It remains unclear why WT1-reactive T cells are not negatively selected in the thymus to maintain central tolerance against self-derived antigens. Intriguingly, a recent study reported that truncated versions of the immunodominant Melan-A/MART1_26-35_ epitope are expressed in human medullary thymic epithelial cells, presumably rendering central tolerance mechanisms incomplete and leading to high frequencies of naive CD8^+^ T cells specific for this antigen in the periphery.^33^ Genome-wide studies are currently ongoing to address this possibility for a variety of other antigens, including WT1.

As the process of clonal deletion to establish central tolerance is TCR affinity-dependent^34^, it is reasonable to assume that WT1-specific T cells engage their target antigens with low or intermediate TCR/pMHC affinities. However, expanded WT1-specific CD8^+^ T-cell cultures displayed functional avidities similar to virus-specific CD8^+^ T cells (see Supplementary material, Fig. S4). In line with this observation, high-affinity donor stem cell-derived WT1_126_-specific CD8^+^ T-cell clones were used for adoptive cell therapy in a recent clinical trial.^35^ Further support for the therapeutic potential of the natural WT1-specific T-cell repertoire is based on immune monitoring studies. Here, the induction of WT1-specific CD8^+^ T-cell responses after allogeneic stem cell transplantation and/or donor lymphocyte infusions demonstrated the immunogenicity of WT1 in cancer patients. Moreover, the induced WT1-specific T cells were probably involved in mediating graft-versus-leukaemia effects.^2^–^4^ Vaccination strategies, which inevitably rely on mobilization of the pre-existing WT1-specific T-cell pool, likewise showed promising results in several clinical studies with response rates up to 63% in haematological diseases (reviewed in ref. 36). We hypothesize that individuals bearing memory WT1-specific T cells in their blood are able to mount stronger WT1-specific responses after vaccination and may therefore benefit most from therapy. It is noteworthy that the techniques described in this study provide compelling tools for improved, highly sensitive immune monitoring in this context.

In our hands, CD8^+^ T cells specific for WT1_37_ were much more abundant than those specific for WT1_126_ regardless of the enrichment process. This observation has implications for the design of therapeutic and immune monitoring strategies, given that many published studies concentrate solely on CD8^+^ T-cell clones specific for WT1_126._ Vaccination studies that used the WT1_126_ epitope alone or in combination achieved only limited T-cell responses in humans.^37^–^39^ In contrast, broader WT1-specific CD4^+^ and CD8^+^ T-cell responses were obtained by approaches incorporating a multitude of WT1-derived epitopes, such as long peptides^40^,^41^ or mRNA-electroporated autologous dendritic cells.^42^ Consistent with these findings, WT1-specific CD4^+^ and CD8^+^ T cells were readily activated *ex vivo* by stimulation with pooled WT1 peptides irrespective of HLA genotype. Collectively, these considerations argue for the therapeutic use of stimuli that cover the whole WT1 protein, thereby taking advantage of the entire pre-existing pool of WT1-specific T cells in healthy individuals.

## Abbreviations

ARTE: antigen-reactive T-cell enrichment
WT1: Wilms’ tumour-1

## Supporting Information

**Figure S1.** Enrichment of WT1-specific CD4^+^ and CD8^+^ T cells on the basis of CD137 expression after stimulation with pooled WT1 peptides. **Figure S2.** Expanded T-cell cultures exhibit WT1-specific cytotoxicity. **Figure S3.***In-silico* database search reveals pathogenic peptides similar to WT1 epitopes. **Figure S4.** Expanded WT1-specific CD8^+^ T cells show similar functional avidities compared to expanded virus-specific CD8^+^ T cells.
