# Supplementary material for: Analysis of the functional WT1-specific T-cell repertoire in healthy donors reveals a discrepancy between CD4+ and CD8+ memory formation
Source: Immunology. 2015 Jun 19;145(4):558–69. doi: 10.1111/imm.12472 (PMC4515135; doi:10.1111/imm.12472)
Supplement: Supplementary file 1 [file imm0145-0558-sd1.pdf]

Supplementary Figure 1

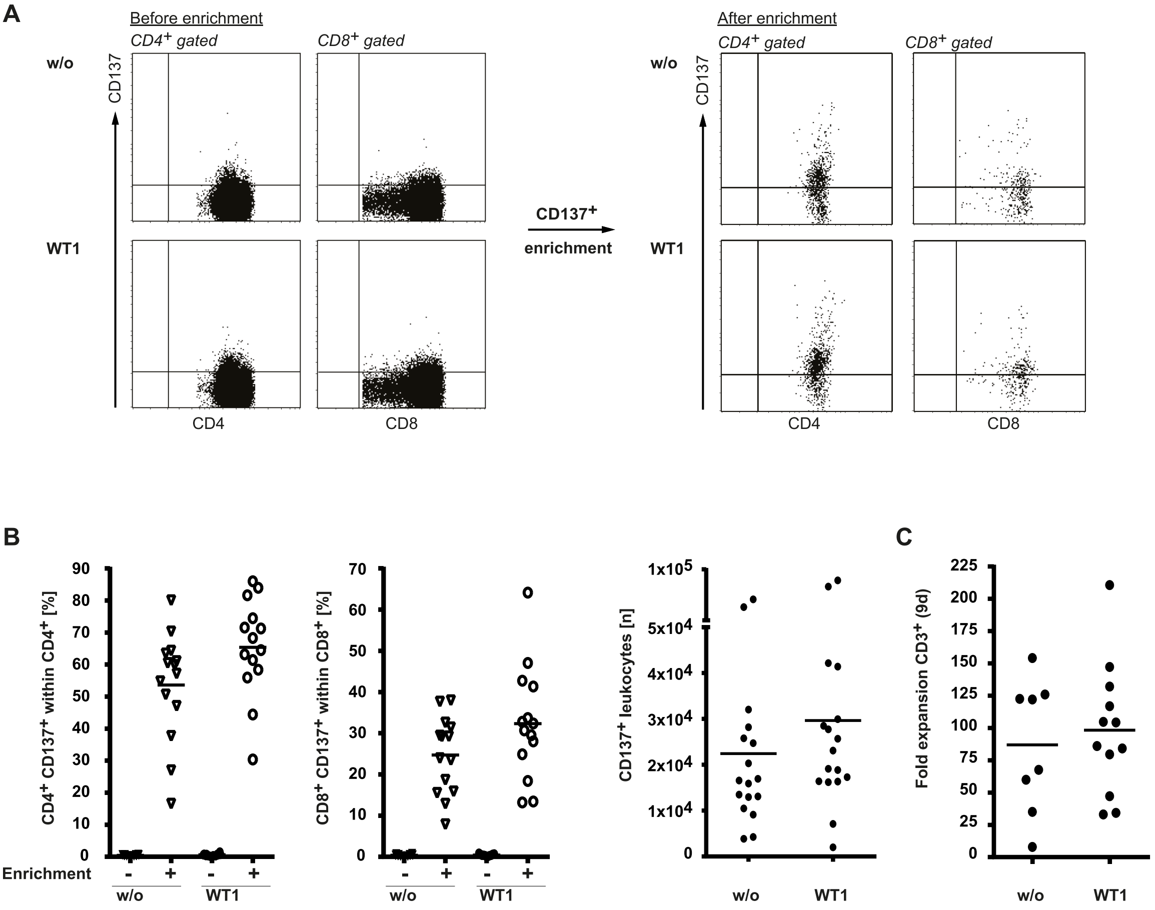

Figure S1. Enrichment of WT1-specific $CD4^+$ and $CD8^+$ T cells on the basis of CD137 expression after stimulation with pooled WT1 peptides. (**A**) Representative dot plots showing CD137 expression on $CD4^+$ and $CD8^+$ T cells incubated with or without pooled WT1 peptides before and after enrichment of $CD137^+$ cells. (**B**) Frequencies of $CD4^+$ $CD137^+$ and $CD8^+$ $CD137^+$ T cells before and after enrichment of $CD137^+$ cells. Right graph: absolute leukocyte numbers after enrichment of $CD137^+$ cells, starting from $1x10^8$ PBMCs. (**C**) Fold expansion of $CD3^+$ T cells within 9 d of culture after CD137 enrichment of unstimulated and WT1-stimulated samples. Significance was determined using the paired Student *t* test. ** $p \leq 0.01$, *** $p \leq 0.001$.

Supplementary Figure 2

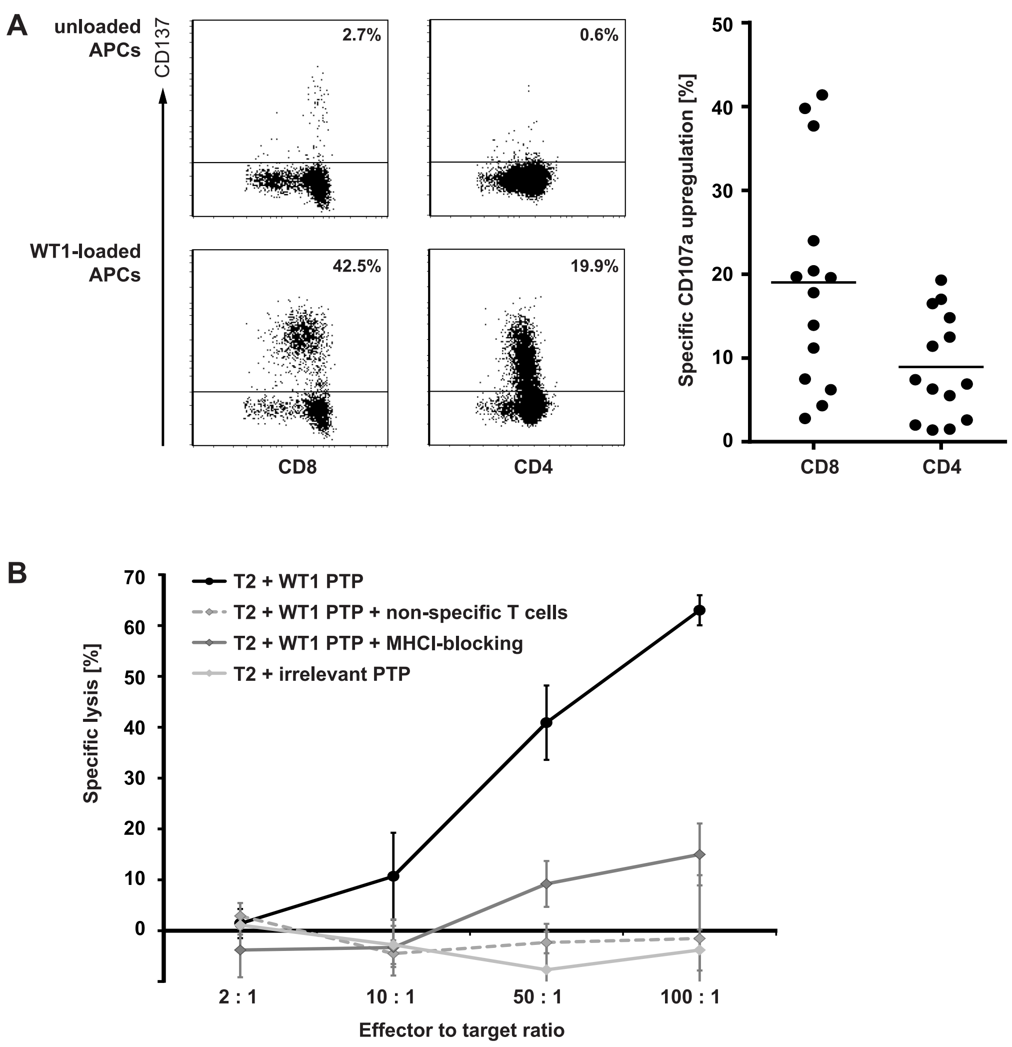

Figure S2. Expanded T cell cultures exhibit WT1-specific cytotoxicity. (**A**) Stimulated, enriched and expanded T cells were co-cultured with autologous APCs, either unloaded or loaded with pooled WT1 peptides. CD107a mobilization on the surface of $CD4^+$ and $CD8^+$ T cells was measured by flow cytometry after 6 h. Dot plots show representative data (left). Summarized results from 14 T cell cultures are shown (right). Background values detected in cultures with unloaded APCs were subtracted. (**B**) Expanded T cells were co-cultured with T2 cells, either unloaded or loaded with pooled irrelevant or WT1 peptides. Specific lysis was calculated by determining the total cell number of living WT1-loaded T2 cells after 4 h co-culture in relation to the total number of unloaded living T2 cells.

Supplementary Figure 3

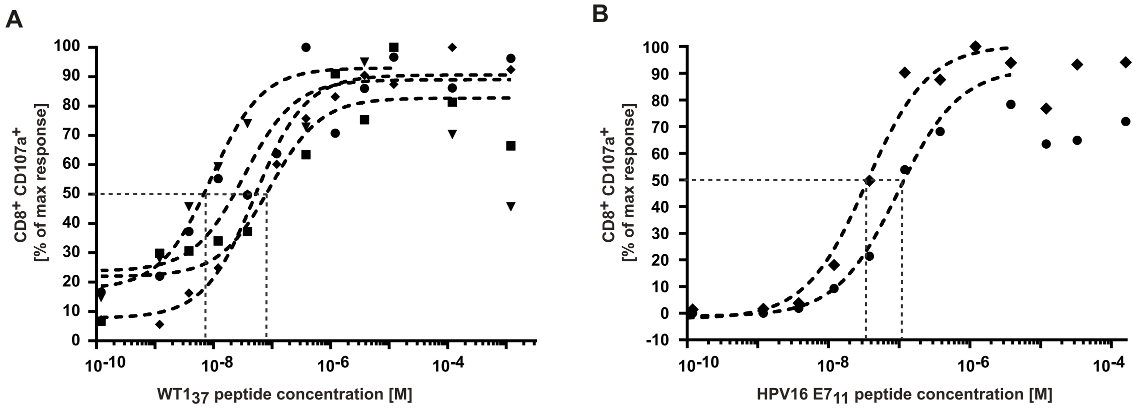

EC_{50} WT137 = 9x10^{-9} to 5x10^{-8} = 9 nM to 50 nM

EC_{50} HPV16 E711 = 3x10^{-8} to 9x10^{-8} = 30 nM to 90 nM

Figure S3. *In-silico* database search reveals pathogenic peptides similar to WT1 epitopes. (**A** and **B**) PBMCs stimulated with either (**A**) pooled WT1 15mer peptides (n = 4 donors) or (**B**) pooled HPV16 E7 15mer peptides (n = 2 donors) were enriched and expanded. T cells were co-cultured with T2 cells loaded with escalating doses of the indicated 9mer peptide. CD107a expression on $CD8^+$ T cells was determined by flow cytometry. Non-linear regression curves and EC_{50} values were calculated using GraphPad Prism.

Supplementary Figure 4

	Protein sequence	Species (Protein)
320-334	KRPFMCAYPGC	Homo sapiens (WT1)
	KKPLMCAYPDC	Aspergillus nidulans (hypothetical protein)

	Protein sequence	Species (Protein)
238-248	WNQMNLGATLK	Homo sapiens (WT1)
	WDKLNLGTATLK	Candida albicans (Chs5p)

	Protein sequence	Species (Protein)
38-48	LDFAPPGASAY	Homo sapiens (WT1)
	LDFTPSEAGAY	Aspergillus kawachii (nuclear envelope core protein)
	LDYTPPSSSEY	Aspergillus niger (unnamed protein)
	LDFAQPGSEISEY	Aspergillus nidulans (hypothetical protein)
	LDFKPRGWKAASAY	Aspergillus ruber (putative oxysterol binding protein)

Figure S4. Expanded WT1-specific CD8$^+$ T cells show similar functional avidities compared to expanded virus-specific CD8$^+$ T cells. BLAST search (http://blast.ncbi.nlm.nih.gov) was performed using three WT1 epitope sequences, which were described to be presented on HLA-DR molecules (Doubrovina, 2012). Search was done against protein databases of Aspergillus sp., Candida sp., and several human viruses (EBV, AdV, VSV, CMV, and Influenza). Selected peptide sequences are shown, predicted HLA-DR anchor amino acids (according to Southwood et al., 1998) in position one and six are marked in green, if additionally conserved to the WT1 peptide sequence amino acids in red.
